# AHCY: A metabolic gatekeeper at the interface of methylation, redox balance, and cellular stress response

**DOI:** 10.1016/j.jbc.2026.111220

**Published:** 2026-02-02

**Authors:** Sarah C. Stanhope, Vikki M. Weake

**Affiliations:** 1 Department of Biochemistry, Purdue University, West Lafayette, Indiana, USA; 2 Purdue Institute for Cancer Research, Purdue University, West Lafayette, Indiana, USA

**Keywords:** *S-*adenosylhomocysteinase (AHCY), *S-*adenosylhomocysteinase hydrolase (SAHH), one-carbon metabolism, enzymatic regulation, methylation potential, redox homeostasis

## Abstract

*S*-Adenosylhomocysteinase (AHCY, also known as SAHH) is a highly conserved enzyme that catalyzes the reversible hydrolysis of SAH into adenosine and homocysteine. As the sole enzyme capable of catalyzing this reaction, AHCY modulates cellular methylation potential required for DNA, RNA, and protein methyltransferase activity. Recent discoveries, however, expand its role well beyond this canonical function, positioning AHCY as a metabolic gatekeeper that integrates one-carbon metabolism with epigenetic regulation, RNA processing, nucleotide balance, and redox signaling. This review brings together mechanistic, structural, and regulatory insights into AHCY while critically evaluating diverse biochemical and biophysical methods for assaying its activity. Comparative structural analyses uncover conserved tetrameric organization alongside species-specific adaptations in oligomeric state, NAD^+^ pocket accessibility, and C-terminal dynamics that shape enzyme catalytic efficiency and regulation. AHCY function is further fine-tuned through a wide spectrum of posttranslational modifications and small-molecule interactions, linking it to transcriptional control, stress adaptation, and viral infection. By linking SAH turnover to methylation capacity and adenosine/homocysteine flux, AHCY coordinates metabolism with chromatin regulation and stress responses. These cross-cutting roles highlight how a single metabolic enzyme bridges catalysis, regulation, and disease. In doing so, AHCY exemplifies the broader principle that metabolic enzymes can have a central role as regulators of metabolic flux and cellular regulation, offering both mechanistic depth and translational promise as a therapeutic target.

## Core pathways of one-carbon metabolism

### One-carbon metabolism: The salvage cycle, methionine cycle, and transsulfuration pathway

One-carbon (1C) metabolism is a fundamental and highly conserved metabolic network that integrates biosynthetic, epigenetic, and redox processes to support cellular homeostasis. To support these physiological processes, 1C metabolism generates and facilitates the transfer of one-carbon units primarily as methyl groups for the synthesis of nucleotides, amino acids, polyamines, and antioxidant molecules. 1C metabolism plays a central role in regulating the methylation status of DNA, RNA, and histones, thereby influencing gene expression, chromatin accessibility, and epigenetic regulation (1, 2).

**Figure 1:**
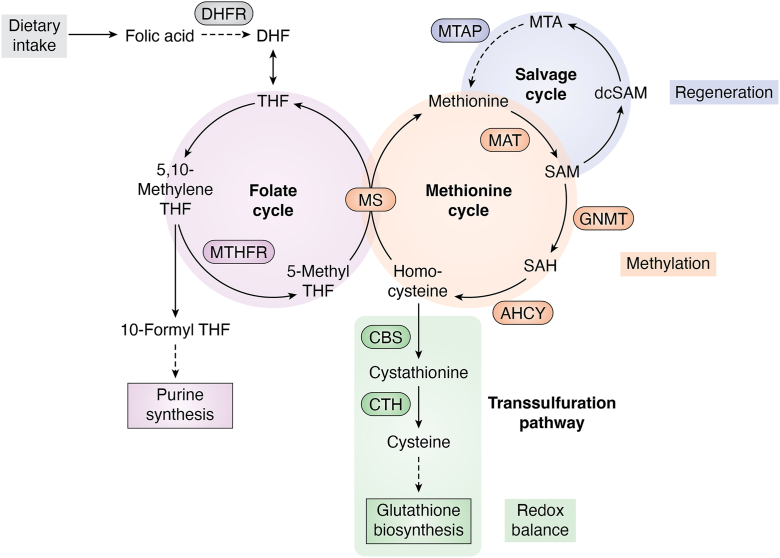
**Overview of folate and one-carbon metabolism**. The folate, methionine, transsulfuration, and salvage pathways form the interconnected branches of one-carbon metabolism. Dietary folic acid is reduced by dihydrofolate reductase (DHFR) to dihydrofolate (DHF) and then tetrahydrofolate (THF), which enters the folate cycle to support nucleotide synthesis and methyl group transfer. The methionine cycle incorporates 5-CH_3_-THF *via* methionine synthase (MS) to regenerate methionine, which is converted by methionine adenosyltransferase (MAT) into *S-*adenosylmethionine (SAM). SAM donates methyl groups to numerous methyltransferases (*i*.*e*., glycine-N-methyltransferase, GNMT), generating the byproduct *S-*adenosylhomocysteine (SAH), which is hydrolyzed by *S*-adenosylhomocysteinase (AHCY) into homocysteine. Homocysteine can be remethylated by MS or directed into the transsulfuration pathway, where cystathionine β-synthase (CBS) and cystathionine γ-lyase (CTH) convert it into cysteine, a precursor for glutathione biosynthesis and cellular redox balance. In parallel, the methionine salvage cycle initiated by methylthioadenosine phosphorylase (MTAP) regenerates methionine from 5′-methylthioadenosine (MTA), a byproduct of polyamine biosynthesis.

The 1C metabolic network is organized into three interlinked branches: the salvage cycle, the methionine cycle, and the transsulfuration pathway (Fig. 1). These pathways are functionally connected and heavily dependent on folate metabolism, which provides the activated one-carbon units. As mammals cannot synthesize folate, it must be taken up *via* dietary intake. Folate-derived cofactors such as 5, 10-methylenetetrahydrofolate (THF) and 5′-methyl-THF act as carriers of one-carbon units and are essential for sustaining the flux through all three branches (1, 3).

The methionine cycle generates and recycles SAM, the universal methyl donor in the cell. SAM is used by methyltransferases to methylate a wide variety of substrates including DNA, RNA, nucleosomes, and proteins. Following methyl group donation, SAM is converted into SAH, which is hydrolyzed by *S*-adenosylhomocysteinase (AHCY; also known as *S*-adenosylhomocysteine hydrolase, SAHH) into homocysteine and adenosine to prevent feedback inhibition of methyltransferases (4). Methionine adenosyltransferase generates SAM from methionine and ATP (1), while methionine synthase, using 5′-methyl-THF and vitamin B12 cofactors, catalyzes the remethylation of homocysteine to produce methionine. SAM abundance is further regulated by glycine-N-methyltransferase, a key enzyme that buffers excess SAM by transferring methyl groups from SAM to glycine, forming sarcosine (5).

The transsulfuration pathway provides a critical link between methylation metabolism and redox balance by converting homocysteine into cysteine. First, cystathionine β-synthase catalyzes the condensation of homocysteine with serine to form cystathionine (6, 7). Then cystathionine is further converted into cysteine by cystathionine γ-lyase (8). This pathway supports glutathione biosynthesis (1, 9), a major antioxidant in the cell; thus, it couples 1C metabolism to redox regulation and oxidative stress responses.

The salvage cycle, also referred to as the methionine salvage pathway or 5′-methylthioadenosine (MTA) cycle, recycles MTA produced during polyamine biosynthesis back into methionine (10). While this pathway varies across organisms, methylthioadenosine phosphorylase is a critical enzyme that initiates MTA breakdown and supports methionine regeneration (10). Importantly, the regenerated methionine can be converted into SAM, thereby sustaining the cellular methyl donor pool required for DNA, RNA, and histone methylation. By linking polyamine metabolism to SAM availability, the salvage pathway provides a crucial buffering mechanism to maintain epigenetic regulation (11).

Together, these three branches coordinate the generation, utilization, and recycling of 1C units to achieve cellular demands for methylation, biosynthesis, and redox defense. 1C metabolism is intrinsically linked to folate availability because the activation and transfer of 1C units rely on folate-derived cofactors. Dysregulation of this network is implicated in a variety of diseases, including cancer, neurodegeneration, and metabolic disorders, highlighting its central role in integrating metabolism with gene regulation and stress adaptation (1, 10, 12).

**Figure 2:**
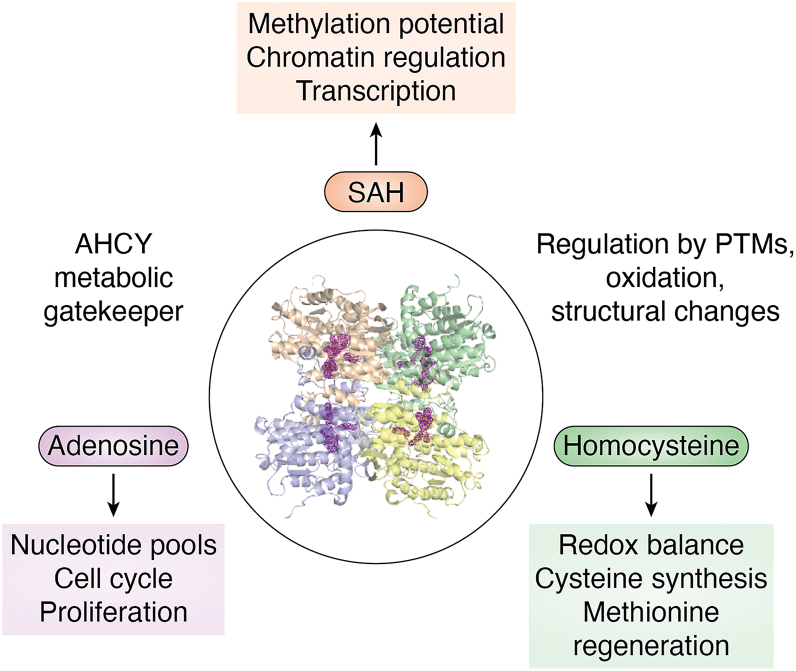
**Conceptual model of AHCY as a metabolic gatekeeper controlling levels of SAH, adenosine, and homocysteine to coordinate diverse cellular processes**. The central structure (AHCY tetramer, PDB: 9PDH) is surrounded by dashed rings representing regulation by posttranslational modifications (PTMs), oxidation, and conformational changes. SAH (*orange*) links to methylation potential, chromatin regulation, and transcription. Adenosine (*purple*) influences nucleotide pools, cell cycle, and proliferation. Homocysteine (*green*) affects redox balance, cysteine synthesis, and methionine regeneration. Together, these pathways illustrate how AHCY couples one-carbon metabolism to gene expression, metabolic homeostasis, and cell fate decisions.

Among these interconnected pathways, AHCY is poised to serve as a regulatory node controlling both methylation potential and flux towards glutathione biosynthesis, positioning it as a key regulator of cellular epigenetic processes, metabolic homeostasis, and redox status (Fig. 2). Because AHCY is the sole enzyme capable of catalyzing the hydrolysis of SAH into adenosine and homocysteine, its activity is required to prevent the accumulation of SAH, a potent inhibitor of methyltransferases. AHCY’s activity is essential for maintaining the methylation capacity of reactions across DNA, RNA, and histones, directly influencing chromatin accessibility and gene expression. In addition, the products of the reaction play distinct roles in cellular regulation. Here, adenosine contributes to nucleotide pool balance and cell cycle control, while homocysteine serves as a critical branch point to the transsulfuration pathway for antioxidant biosynthesis. By maintaining the balance of SAH and downstream products, AHCY integrates 1C metabolism to broader processes including methylation capacity, redox homeostasis, and cellular stress responses. This review will provide a perspective on AHCY’s canonical activities and structure-function relationships to emphasize its central role in cellular regulation.

## Mechanistic insights into AHCY catalysis and metabolic control

### AHCY catalytic mechanism

**Figure 3:**
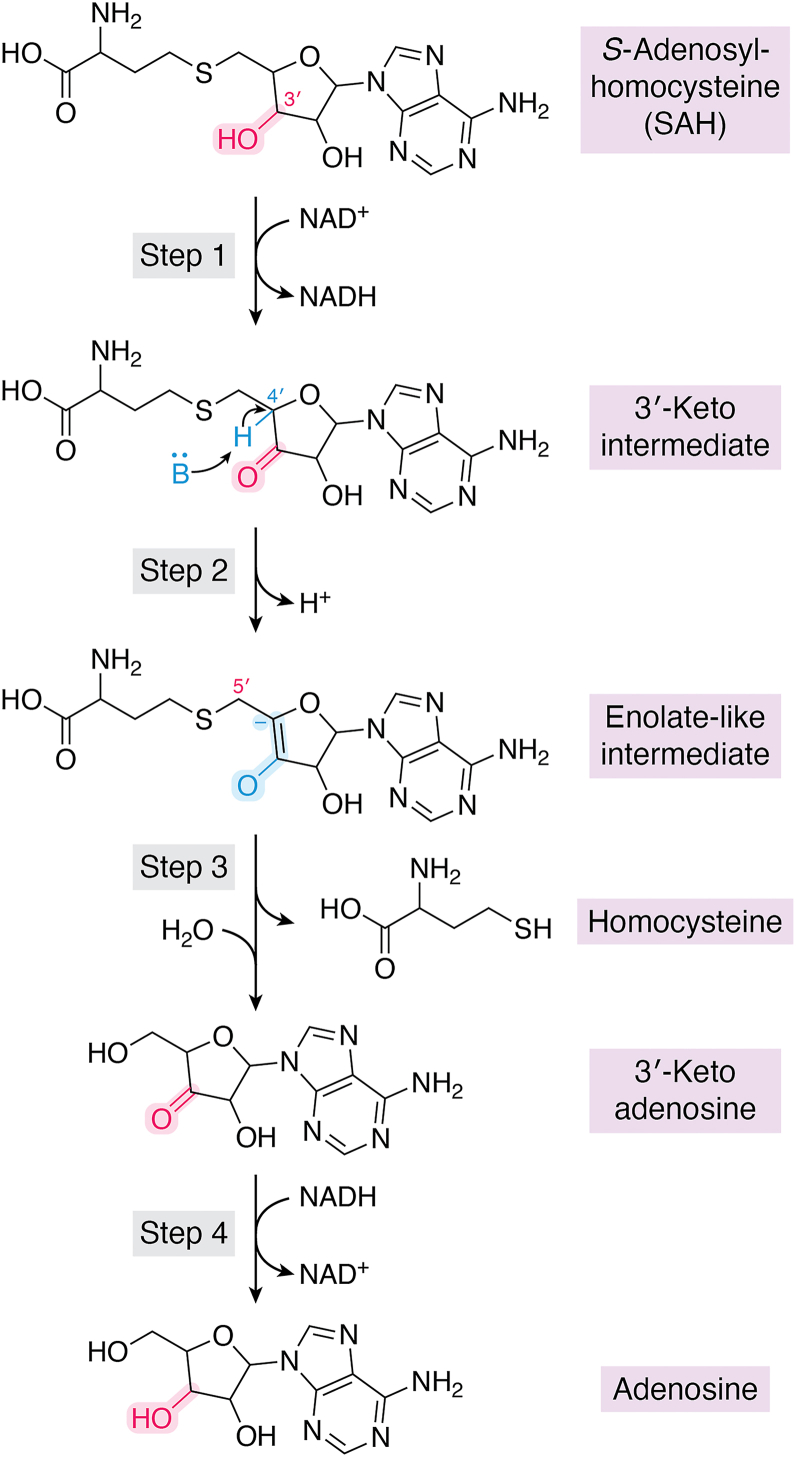
**Catalytic mechanism of AHCY**. The NAD^+^ -dependent hydrolysis of SAH proceeds through 4 redox-driven steps. *Step 1*: SAH binds in the substrate-binding domain, inducing a conformational shift that closes the enzyme *via* hinge rotation and positions the substrate adjacent to the NAD^+^ cofactor. The 3′-hydroxyl group of the ribose ring is oxidized to a 3′-keto intermediate, with concurrent reduction of NAD^+^ to NADH, generating a reactive electrophile. *Step 2*: The increased acidity of the adjacent C4′-hydrogen allows abstraction by a catalytic base, producing a carbanion stabilized as an enolate-like intermediate. *Step 3*: The enolate-like intermediate undergoes β-elimination, cleaving the C5′–S bond and releasing homocysteine, leaving a 3′-keto-adenosine intermediate. *Step 4*: The carbonyl group of 3′-keto-adenosine is reduced by NADH to form adenosine, regenerating NAD^+^ and allowing the enzyme to return to its open conformation. Key reactive centers are highlighted in *magenta* and *blue*.

**Figure 4:**
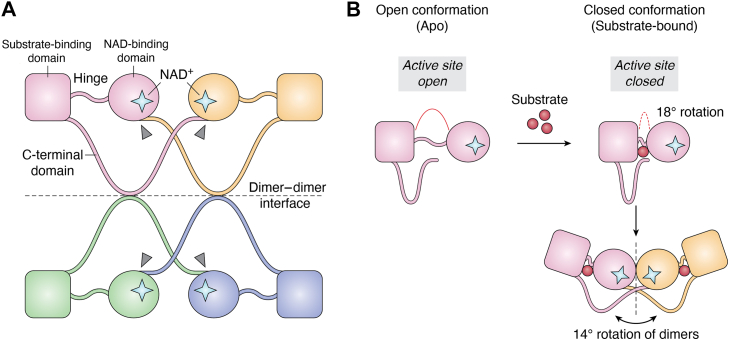
**Quaternary assembly and conformational dynamics of AHCY**. *A*, tetrameric arrangement of AHCY subunits 1 *(pink)*, 2 *(orange)*, 3 *(green)*, and 4 *(blue)*. Each subunit contains a substrate-binding domain (*square*) and an NAD-binding domain (*circle*). NAD^+^ molecules are indicated as stars. The hinge region and C-terminal tails are labeled, with arrows (*gray*) marking the extension of each C-terminal tail into the adjacent subunit’s NAD-binding domain at the dimer–dimer interface. *B*, conformational change of AHCY upon substrate binding. In the open (apo) conformation (*left*), the substrate-binding and NAD-binding domains are separated, leaving the active site accessible. Substrate binding (*red*) induces an 18° rotation between the two domains, resulting in a closed conformation (*right*). This closure is followed by a 14° rotation of the dimer relative to its partner.

The catalytic mechanism of AHCY first proposed by Palmer and Abeles *et al*. and supported by Yamada *et al*.*,* is a redox-driven enzymatic sequence coordinated by the bound NAD^+^ cofactor where SAH elimination is followed by the release of adenosine and homocysteine (Fig. 3) (13, 14, 15). First, SAH binds to the active site in the substrate-binding domain inducing a conformational shift. SAH binding enables the transition of the protein to its closed conformation *via* rotation of the hinge region, bringing the substrate-binding and cofactor-binding domains into close proximity (Fig. 4) (15). Once SAH is bound and positioned near the NAD^+^ cofactor, the 3′-hydroxyl group of the ribose ring is oxidized to a 3′-keto intermediate while simultaneously reducing NAD^+^ to NADH (*Step 1*) (13, 14). This transition from an alcohol to a ketone oxidation moiety produces a reactive electrophilic intermediate enhancing the acidity of the adjacent C4′-hydrogen. The acidification of the C4 on the ribose ring enables its abstraction by a base within the active site (*Step 2*), which is the rate-limiting step in the mechanism (15). The resulting carbanion is stabilized producing an enolate-like intermediate. The enolate-like intermediate undergoes an elimination step leading to cleavage of the C5′-S bond followed by the release of homocysteine yielding the 3′-keto-adenosine intermediate (*Step 3*) (13, 14). Lastly, the carbonyl group of 3′-keto-adenosine is reduced by NADH forming adenosine (*Step 4*) (13, 14). Concurrently, the NADH is re-oxidized to NAD^+^, enabling the enzyme to return to an open conformation to support further catalytic cycles (13, 14).

AHCY’s reaction is reversible where the catalysis of SAH is termed the hydrolytic direction and the production of SAH is known as the synthetic direction (14). The synthetic direction is favored under *in vitro* conditions (14, 15). However, under physiological conditions, the equilibrium is pushed toward the hydrolytic direction for two main reasons. First, SAH is rapidly catabolized to prevent the inhibition of methyltransferases (16). SAM and SAH operate as a ratio where SAM is substantially more abundant than SAH *in vivo*. For example, healthy human plasma exhibits SAM concentrations of ∼120 nM compared to SAH at ∼21 nM (SAM:SAH ≈ 5.6:1) (17). This high SAM:SAH ratio supports effective methylation potential. Second, homocysteine is in high demand by the cell and serves as a branchpoint from the methionine cycle to the transsulfuration pathway (18). Thus, breakdown of SAH and rapid removal of homocysteine and adenosine push the equilibrium toward the hydrolytic direction. Proper flux through this pathway supports both methionine regeneration and glutathione biosynthesis, which aids in maintaining redox homeostasis.

### Methods of measuring AHCY activity

There are several published and widely used methods for measuring AHCY activity in both the hydrolytic and synthetic direction. The most common assays for measuring AHCY activity include spectrophotometric-, fluorescence-, radiometric-, high performance liquid chromatography- (HPLC), and mass spectrometry (MS)-based assays. Over the years, there have been extensive efforts to develop MS-based methods to enable sensitive, high-throughput characterization methods. These biochemical methods have enabled the determination of AHCY kinetic parameters, revealing low micromolar substrate affinities (*K*_*m*_) and high catalytic efficiencies (*k*_*cat*_*/K*_*m*_) consistent with its role in maintaining low intracellular SAH levels (Table 1).

**Table 1:** Species-specific AHCY kinetic parameters

AHCY species	Analysis methods	K_M_ (μM)	*k*_*cat*_ (s^-1^)	*k*_*cat*_/K_M_ (× 10^-3^ μM^-1^ s^-1^)	*v*_*max*_ (× 10^-3^ μM s^-1^)	K_i_ (μM)	Ref
*Drosophila melanogaster*	HT-DESI-MS	44 ± 11	0.34 ± 0.07	8 ± 2	10 ± 2	29 ± 13	(29)
*Caenorhabditis elegans*	HT-DESI-MS	25 ± 5	0.13 ± 0.01	5 ± 1	3.9 ± 0.4	110 ± 19	(29)
*Homo sapiens*	RapidFire MS	60.5	-	-	-	-	(26)
*Homo sapiens*	Spectrophotometric	29 ± 4	1.63 ± 0.07	57 ± 8	330 ± 10	-	(30)
*Homo sapiens*	HPLC	7.8 ± 0.2	0.530 ± 0.008	68 ± 2		-	(31)
*Plasmodium falciparum*	HPLC	1.2 ± 0.1	0.0233 0.0003	19 ± 2		-	(31)
*Leishmania donovani*	HPLC and Spectrophotometric	21 ± 3	0.48 0.05	23 ± 4	-	-	(32)
*Bradyrhizobiumelkanii*	Photometric	41 ± 5	-	-	417 ± 20	-	(19)

Table describing *in vitro* AHCY kinetic parameters across species with analysis method specified.

Both spectrophotometric and fluorescence-based assays measure AHCY activity through detection of product formation by thiol conjugation. Traditionally, these assays utilize a coupled enzyme reaction typically involving the addition of adenosine deaminase or a thiol-modifying compound such as 5,5′-dithiobis(2-nitrobenzoic acid) (DTNB) (19, 20). The coupling of a reaction with adenosine deaminase enables the deamination of adenosine into inosine, which is then quantified by measuring the decrease in absorbance at 265 nm (19, 20). In the case of thiol conjugation using DTNB, reaction with homocysteine releases 2-nitro-5-thiobenzoic acid and is quantified at 412 nm (19, 20). A major pitfall for assays utilizing DTNB is that it is not specific to the thiol group of homocysteine and therefore can react with any thiol group including AHCY itself (21). For example, AHCY can be modified by DTNB at three cysteine residues, leading to inactivation of the enzyme (21). Fluorescence-based assays rely on a similar principle of thiol conjugation but employ thiol-reactive fluorescent dyes such as ThioGlo1, which yield a fluorescent product at 510 nm upon binding homocysteine (22). Fluorescence-based assays can be used for real-time measurements of AHCY kinetic activity, providing an advantage compared to endpoint-based spectrophotometric assays.

Radiometric and HPLC-based assays both quantify AHCY activity by directly detecting reaction products rather than relying on secondary coupling reactions. Radiometric assays use [^3^H]- or [^14^C]-labeled SAH (13), with AHCY-mediated cleavage producing labeled adenosine and homocysteine that can be separated from the substrate by thin-layer chromatography or ion-exchange columns (23, 24). These approaches offer excellent sensitivity and precise kinetic measurements but require specialized equipment and strict radioactive safety protocols. In contrast, HPLC assays achieve product detection through chromatographic separation of SAH, adenosine, and homocysteine, with UV absorbance used to monitor substrate consumption or adenosine formation (25). Although HPLC requires careful optimization of mobile phase and column conditions, it provides highly reproducible, quantitative, and high-resolution measurements, making it valuable for time-course analyses.

In recent years, MS-based assays have been the preferred method for characterizing AHCY activity as they require small amounts of recombinant protein, are highly sensitive, and are suitable for high-throughput studies. Uchiyama *et al*. developed one of the first high-throughput, label-free assays for measuring AHCY hydrolytic activity using RapidFire-MS (26). This plate-based assay is able to quantify both unmodified substrate and products by identifying their mass-to-charge ratio and can be modified for use in cells and/or recombinant protein. Similar to RapidFire-MS, we developed a high-throughput desorption electrospray ionization mass spectrometry label-free assay to directly quantify adenosine production from complex bioassay mixtures (27, 28, 29). A major benefit of this technique is that high-throughput desorption electrospray ionization mass spectrometry is a contactless ambient ionization that enables direct analysis of samples (27, 28). This method can be used for common biochemical buffers with high concentrations of nonvolatile salts and detergents (27, 28). These assays enable determination of key kinetic parameters including *K*_*m*_, *k*_*cat*_, *k*_*cat*_*/K*_*m*_, *V*_*max*_, and *K*_*i*_.

## The molecular architecture of the AHCY homotetrameric complex

### Conserved AHCY domains

**Figure 5:**
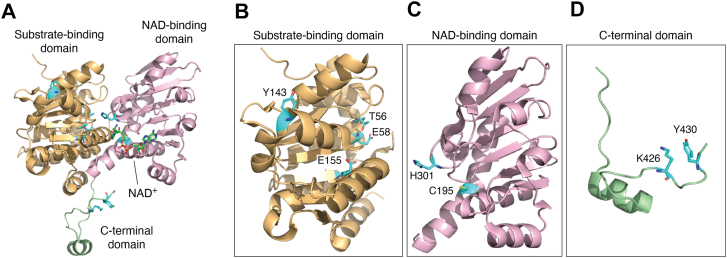
**Critical residues within human AHCY** (PDB: 1LI4). *A*, domain architecture of human AHCY monomer with the substrate-binding domain (*orange*), NAD-binding domain (*pink*), and C-terminal domain (*green*) indicated. Bound NAD^+^ is shown in *green* inside of NAD-binding domain. *B*, substrate-binding residues T56, E58, and E155 stabilize the catalytic pocket. Y143 contributes to structural and thermal stability. *C*, NAD-binding domain residues H301 and C195 are positioned near the NAD^+^ pocket, enabling domain closure and redox-sensitive modulation of activity. *D*, C-terminal domain residues K426 and Y430 are at the dimer–dimer interface and extend into the adjacent subunit to stabilize NAD^+^ binding and support tetramer formation.

Human AHCY functions as a homotetramer and each subunit contains a substrate-binding domain (residues M1-N181 and G352-L402), two hinge regions (residues D182-R196 and G352-P354), a cofactor-binding domain (residues D182-M351), and a C-terminal tail (residues N403-Y431) (Fig. 5, *A*–*D*) (33, 34, 35). The monomers associate with one another through noncovalent interactions forming a dimer of dimers configuration to create the full tetramer. AHCY undergoes conformational changes upon substrate binding and product release (33, 35). The open conformation is characterized by the absence of substrate but the presence of bound NAD^+^. This conformation appears as a large cleft between the substrate- and cofactor-binding domains. When substrate is bound, the hinge regions rotate ∼18° leading to closure of the cleft, bringing the two domains together followed by a ∼14° rotation of the dimers (35). Upon release of the adenosine product, the protein transitions back to the open conformation (33, 36) (Fig. 4).

The substrate-binding domain is the largest domain containing over 200 amino acids forming 8 α-helices and 8 β-strands (34, 35) (Fig. 5*B*). This domain is located on the external surface of the tetramer far from the core of the protein and plays little to no role in the interaction between subunits (33, 34). The lack of interaction among the substrate-binding domains enables flexibility upon substrate binding (35). The adenosine moiety of SAH is nestled in a hydrophobic pocket and is stabilized *via* hydrophobic interactions and hydrogen bonding while its homocysteine moiety extends into a channel that is created upon binding of the substrate (33, 35).

The cofactor-binding domain is the second largest domain with 155 residues arranged into a Rossmann fold motif, which is one of the most common tertiary structural domains in proteins that bind nucleotides such as NAD^+^ (33, 34, 37) (Fig. 5*C*). This structural motif is characterized by a β-sheet flanked by α-helices enabling efficient NAD^+^ binding and electron transfer to support catalysis. This fold supports the formation of an NAD^+^-binding pocket where each monomeric subunit contains one molecule of tightly but not covalently bound, NAD^+^. Previous crystal structure studies have determined that a minimum of a dimer conformation is needed to properly form the NAD^+^-binding sites (34). The NAD^+^ molecule is bound in a rigid pocket rendering the cofactor-binding domain less flexible than the substrate-binding domain (35). This precise positioning of the NAD^+^ molecule coordinates redox reactions (NAD^+^ to NADH) and assists in product release after catalysis.

The C-terminal tail region is much smaller in size compared to the other two conserved domains, but it plays a critical role in tetramer formation and coordination of the subunits (33, 34) (Fig. 5*D*). This C-terminal region consists of fewer than 30 amino acid residues that form a helix-loop-helix structure. This structural element extends into adjacent subunits to support multimerization and participates in forming the adenosine pocket (33). Formation of a tetramer creates a channel through the center of the protein built in part by the 4 sets of helices from the cofactor-binding domain and the tails of the C-terminal domains (34). This channel runs along the long axis of the tetramer and aligns with the C-terminal domains of each subunit with the tails extending into the center (35). The interior surface of the channel is covered by thirteen hydrophilic residues. This channel does not play a role in catalysis but instead provides structural integrity to the tetramer, facilitating its transition between the open and closed conformations (36).

The two hinge regions (*i*: *D*182-R196; *ii*: G352-P354) connect the substrate-binding domain to the cofactor-binding domain (33, 35). The first hinge region (182–196) connects the C-terminal end of the substrate-binding domain to the N-terminal of the cofactor-binding domain, enabling rotation toward the substrate-binding domain. The second hinge region (G352-P354) connects the end of the cofactor-binding domain to the C-terminal portion of the substrate-binding domain, stabilizing the tetramer as it undergoes conformational changes (33, 35).

### Structural divergence of AHCY across species

**Figure 6:**
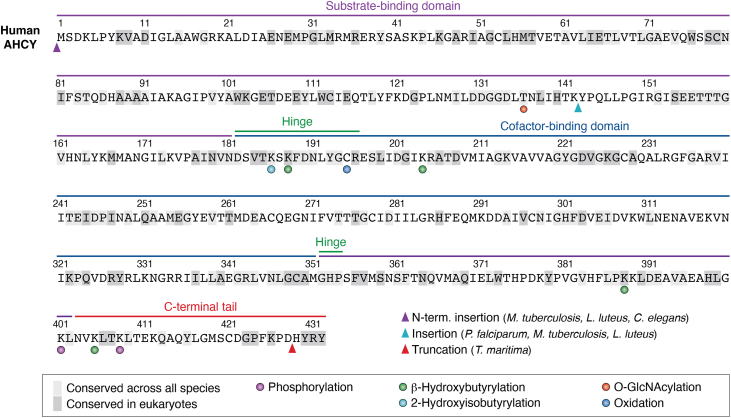
**Sequence alignment of human AHCY with conservation and posttranslational modifications indicated**. Conservation was determined from a multiple sequence alignment of AHCY homologs from 8 species: *Homo sapiens*, *Rattus rattus*, *Caenorhabditis elegans*, *Drosophila melanogaster*, *Thermotoga maritima*, *Mycobacterium tuberculosis*, *Lupinus luteus*, and *Plasmodium falciparum*. Residues highlighted in *light gray* are absolutely conserved across all 8 species, whereas *dark gray* indicates residues conserved among eukaryotic organisms only. Domain architecture is shown above the alignment: substrate-binding domain (*purple*), cofactor-binding domain (*blue*), hinge regions (*green*), and C-terminal tail (*red*). Experimentally reported posttranslational modifications in human AHCY are annotated: phosphorylation (*purple circle*), O-GlcNAcylation (*red circle*), 2-hydroxyisobutyrylation (*green circle*), 2-hydroxyisobutyrylation (*light blue circle*), and oxidation (*dark blue circle*). Insertions and truncations between species are indicated within the alignment by colored arrows.

While the overall structure of AHCY is highly conserved, species-specific adaptations have been observed *via* high-resolution structures that influence oligomeric state, domain organization, and cofactor accessibility (Fig. 6). These structural variations provide insight into AHCY’s functional divergence that may potentially be exploited for selective inhibitor development.

In mammals such as humans (PDB: 1LI4) and rats (PDB: 1B3R), AHCY crystallizes as a homotetramer in a dimer-of-dimers configuration. The stability of the tetramer is mediated through a conserved C-terminal helix-loop-helix motif and NAD^+^-binding domain interfaces (33, 34). This quaternary assembly enables domain mobility necessary for substrate turnover (33, 34). In plants, such as *Lupinus luteus*, AHCY (PDB: 3OND) assembles as a stable homodimer, indicating that tetramerization is not universally required for activity and may be dispensable in certain eukaryotes (38).

The C-terminal tail encompassing the helix-loop-helix motif observed in mammalian AHCY plays a direct role in forming the adenosine-binding pocket and modulating NAD^+^ dynamics (33, 34). Interestingly, *Thermotoga maritima* AHCY (PDB: 3X2E) is truncated at the C terminus resulting in the deletion of conserved residues including lysine K425 and tyrosine Y429 and is observed to crystallize as a dimer or a loosely associated tetramer (39). This truncation leads to an incomplete NAD^+^ pocket that is partially solvent-exposed, which promotes higher NAD^+^ turnover but also increases cofactor dissociation (39). Additionally, the truncated structure shows enhanced thermostability, which may be an adaptation to the thermophilic environments that *T*. *maritima* is accustomed to (39). In humans, the integrity of the C-terminal region appears to be essential for AHCY’s assembly as missense mutations affecting the C-terminal loop structure result in a destabilized tetramer and a clinical AHCY deficiency (40).

The architecture of the NAD^+^-binding pocket varies greatly across species. In mammals, the cofactor is buried within the core of the enzyme interacting with residues from both monomeric subunits at the dimer interface. Here, the NAD^+^ pocket is shielded, which likely evolved to minimize uncontrolled redox fluctuations (33, 34). In contrast, bacterial homologs including *T*. *maritima* and *Mycobacterium tuberculosis* show a partially exposed NAD ^+^-binding pocket due to domain truncations or altered hinge dynamics (36, 39). Although NAD^+^ accessibility is enhanced in these structures, decreased catalytic efficiency is also observed (36, 39). Lastly, *Pseudomonas aeruginosa* AHCY possesses both K^+^ and Zn^2+^ ions near the active site that aid in modulating NAD^+^ binding and catalytic activity (41).

Although the overall domain architecture of AHCY is well conserved, there are species-specific alterations in loop insertions and solvent accessibility that have functional implications. For example, in *Plasmodium falciparum* (PDB: 1V8B), there are insertions in the catalytic domain that alter the charge distribution (41). These features of AHCY may modulate protein–protein interactions or regulation *via* posttranslational modifications.

### Critical residues for AHCY structure, stability, and activity

The structural integrity of AHCY relies on conserved residues for proper assembly, cofactor and substrate binding, and to facilitate the transition between different conformational states. Turner *et al*. published the first crystal structure of human AHCY (PDB: 1A7A) identifying that K426 and Y430 from one monomeric subunit interact with the bound NAD^+^ of the adjacent monomer *via* hydrogen bonding with the sugar hydroxyls of NAD^+^ (33) (Fig. 5*D*). This indicates that NAD^+^ binding requires at least a dimeric form of AHCY. Site-directed mutagenesis of an aspartic acid at position 244 to a glutamic acid (D244E) in AHCY led to disrupted hydrogen bonding with K425 (position 426 in human AHCY) and NAD^+^ (34). Additionally, this mutation induced a conformation shift from an open to a closed state, resulting in a disordered C-terminal domain, and decreased bound NAD^+^ content (34). WT AHCY binds 1 mol of NAD^+^ per subunit (42), whereas the D244E mutant contained only 0.05 mol of NAD^+^ (34).

Key AHCY residues required for the binding and release of substrate have also been identified. Three residues central to SAH binding include threonine 56 (T56), glutamate 58 (E58), and E155 based on the studies of AHCY from rat liver (34) (Fig. 5*B*). T56 and E58 form hydrogen bonds with the adenine ring of SAH, while E155 hydrogen bonds with ribose moiety of SAH (34). These hydrogen bond interactions maintain the architecture of the catalytic pocket. Similarly, in both human and mouse AHCY, there is a repositioning of histidine 301 (H301) in response to SAH binding (35). Upon SAH binding, H301 flips out of the binding pocket facing the solvent and displaces the 5′OH group of the adenine ring to accommodate the homocysteine moiety and coordinates a catalytic water molecule (35). Kusakabe *et al*., were the first to report the presence of an Na^+^ cation in the active site near the hinge region in close proximity to adenosine (35). Na^+^ cations have been demonstrated to enhance both substrate binding and catalytic activity, in part by interactions with H353, which in turn recognizes the adenine ring *via* hydrogen bonds. In *M*. *tuberculosis*, H363 rotates upon SAH binding to create an access channel for homocysteine release (36). These residues work together to stabilize domain interfaces and ensure both substrate and cofactor accessibility.

Protein stability under thermal stress is also influenced by specific residues in AHCY as observed in *AHCY-*deficient patients with an amino acid substitution at position 143 from a tyrosine to a cysteine (Y143C) in the catalytic domain (Fig. 5*B*),which experience severe biochemical abnormalities such as elevated homocysteine levels in plasma and disrupted SAM to SAH levels (40). This base substitution disrupted hydrogen bonding within a helical region, resulting in reduced AHCY expression and enhanced thermo-sensitivity. At elevated temperatures, the Y143C variant lost structural integrity more rapidly than WT and was unable to refold (40). Y143 is thought to contribute to secondary structure and stability most likely through intramolecular hydrogen bonding that anchor nearby helices.

The catalytic efficiency of AHCY is dependent on residues that facilitate substrate conversion into products and redox cofactor recycling. As discussed previously, K426 is required for proper NAD^+^ positioning and K426R mutants in human AHCY lacked catalytic activity (43). The K426R form of AHCY was isolated in its NADH-bound form, indicating that it retained oxidative activity, but lacked the 5′-hydrolytic activity that is necessary to complete the catalytic cycle (43). Unlike the K426R mutant that still formed a tetramer, other mutants such as glutamic acid (K426E) and alanine (K426A) disrupted quaternary structure and produced monomers that did not bind NAD^+^ nor NADH (43). Thus, K426 has important roles in both redox cofactor recycling and quaternary structure.

Several cysteine residues in AHCY are also important for its catalytic activity. Yuan *et al*. identified several cysteine residues per subunit that were available to be chemically modified, leading to inactivation of AHCY when the protein was in an open conformation with no bound substrate (21). Notably, one of the identified cysteines, C195, was identified as crucial for enzymatic activity. Mutations of C195 to a serine (C195S) or an aspartic acid (C195D) led to a significant reduction in enzyme activity with *k*_*cat*_ values decreasing to about 12% and 7% of the WT, respectively (21). Despite these reductions, the mutants maintained the ability to catalyze both the 3′-oxidative and 5′-hydrolytic reactions (21). Additionally, neither mutation altered secondary structure (21). Supporting these findings, our recent study in *Drosophila melanogaster* AHCY highlighted C195 as being necessary for enzymatic activity *in vitro* (29) (Fig. 5*C*). We observed a substantial decrease in the *K*_*m*_ followed by a 4-fold decrease in the catalytic efficiency (*K*_*cat*_*/K*_*m*_*)* of the C195S mutant, indicating a higher affinity for the substrate with decreased product output (29). These studies support the idea that C195 plays a pivotal role in the catalytic mechanism by potentially influencing the 3′-reduction potential and facilitating product release.

### Enzyme kinetic comparison between species

Across species, AHCY exhibits notable differences in kinetic parameters, which suggests divergent evolutionary needs shaping enzyme performance and regulation to meet cellular demands. The observed differences likely indicate species-specific needs in balancing efficient SAH catabolism and the need for dynamic regulation of methylation potential in diverse cellular environments. It is important to note the caveat that differences in methodology, experimental design, and conditions between studies limit the direct comparability of these measurements (Table 1).

Mammalian AHCY generally exhibits moderate substrate affinity (*K*_*m*_) values ranging from 7.8 to 60.5 μM, supporting that SAH is efficiently catabolized at relevant physiological concentrations (26, 30). Along with *K*_*m*_, mammalian AHCY displays high catalytic efficiencies (*k*_*cat*_*/K*_*m*_) from 57 to 68 x 10^-^^3^ μM^-1^ s^-1^, indicating a strong substrate affinity coupled with product turnover (30). Moderate *K*_*m*_ values coupled with high catalytic efficiency enables the fine-tuning of cellular methylation capacity to adjust to metabolic signals. The buried NAD^+^ pocket and stabilized tetrameric structure observed in mammalian AHCY likely contributes to this tight catalytic precision (33, 34).

In invertebrates such as *Drosophila*, AHCY shows a higher *K*_*m*_ (44 ± 11 μM) and a lower catalytic efficiency (8 ± 2 × 10^-3^ μM^-1^ s^-1^) in comparison to mammals, which suggests a reduced substrate affinity and turnover rate (29). Notably, the redox-sensitive C195S mutant further decreases catalytic efficiency (2.1 ± 0.4 × 10^-3^ μM^-1^ s^-1^) and increases inhibition constants (*K*_*i*_), which has also been observed in mammalian AHCY mutants (21). These studies support a role for C195 in the redox regulation of enzyme activity (21, 29). These findings imply that *Drosophila* AHCY may operate under more flexible catalytic constraints. In *Caenorhabditis elegans*, AHCY exhibits *K*_*m*_ values similar to *Drosophila* but with even lower catalytic efficiencies (5 ± 1 × 10^-3^ μM^-1^ s^-1^) and high *K*_*i*_ values (110 ± 19 μM), suggesting greater susceptibility to feedback inhibition by SAH (29). Thus, there may even be differences in methylation dynamics and regulation between invertebrates such as *C*. *elegans* and *Drosophila*.

AHCY from protozoa and bacteria demonstrates distinct kinetic adaptations than its mammalian counterpart. *P*. *falciparum* AHCY displays a very low *K*_*m*_ (1.2 ± 0.1 μM) and a moderate catalytic efficiency (∼19 × 10^-3^ μM^-1^ s^-1^), suggesting strong substrate affinity enabling rapid clearance of SAH (41). Similarly, *Leishmania donovani* AHCY exhibits intermediate *K*_*m*_ (21 μM) and catalytic efficiency (∼23 × 10^-3^ μM^-1^ s^-1^), emphasizing efficient SAH catabolism under fluctuating conditions (32). Bacterial AHCY are less well characterized than eukaryotic homologs with the exception of *Bradyrhizobium* *elkanii* AHCY. *B*. *elkanii* AHCY displays a *K*_*m*_ of 41 ± 5 μM, which is higher than most mammalian enzymes, suggesting lower substrate affinity. Despite this, it supports SAH turnover within bacterial methylation cycles (19).

In summary, while the core AHCY mechanism is conserved, species-specific differences in *K*_*m*_, *K*_*i*_, and catalytic efficiency highlight variations in SAH turnover kinetics that likely support each organism’s metabolic and regulatory requirements. Structural features such as oligomeric state, NAD^+^ pocket accessibility, and C-term tail domain flexibility most likely influence functional adaptation.

### AHCY-like noncanonical protein family members

AHCY has two noncanonical paralogs termed AHCY-like 1 (AHCYL1) and AHCY-like 2 (AHCYL2) (44). These proteins have been identified in multicellular organisms such as vertebrates and arthropods, but not in plants (45). AHCYL1 and AHCYL2 contain an N-terminal inositol 1,4,5-trisphosphate receptor (IRBIT) domain and a C-terminal AHCY domain. AHCYL2 also contains a unique, nonstructured N-terminal region that contains a high percentage of proline and alanine residues (44). Although there is high sequence homology in the AHCY domain between AHCYL1 and AHCYL2, there are substitutions at critical catalytic residues in the paralogs rendering both inactive and unable to perform SAH catalysis (44).

The addition of the IRBIT domain suggests AHCYL1/2 are evolutionary distinct from AHCY and likely have other biological functions. One of the best characterized functions of AHCYL1 is modulating intracellular calcium (Ca^2+^) signaling. AHCYL1 utilizes its IRBIT domain to bind inositol 1,4,5-trisphosphate receptors on the endoplasmic reticulum, competing with inositol 1,4,5-trisphosphate (46, 47). This binding event inactivates the Ca^2+^ channel to prevent the release of Ca^2+^, thus modulating the activity of Ca^2+^ channels. Although AHCYL2 has not been extensively studied and has no documented role in Ca^2+^ calcium signaling, studies hypothesize that it also modulates Ca^2+^ channels *via* its PEST (proline (**P**), glutamic acid (**E**), serine (**S**), and threonine (**T**)) and IRBIT domains similar to AHCYL1 (46).

AHCYL1 and AHCYL2 also have well-documented roles in ion transport. For example, AHCYL1 and AHCYL2 localize throughout renal tubules in the kidney to support and manage acid levels by regulating ammonium ion (NH_4_^+^) transport (48). The AHCYL proteins do not transport this ion directly; instead, data suggest they enhance the activity of key bicarbonate transporters by binding to and displacing inhibitory regions, thereby activating transporters such as the sodium bicarbonate cotransporter 1 (NBCn1) and NBCn2 (46). This interaction promotes the influx of bicarbonate ions to neutralize and convert NH_4_^+^ into a form that can be reabsorbed in the kidney. The regulatory roles of AHCYL1 and AHCYL2 in cellular signaling and ion transport emphasize their functional divergence from AHCY, highlighting a distinct role for this protein family beyond 1C metabolism and methylation processes.

Although most studies have demonstrated roles outside of metabolism for AHCYL1 and AHCYL2, a review by Devogelaere *et al*. postulated that AHCYL1 could regulate AHCY directly through scaffolding or localization, possibly forming a heterotetramer (44). Supporting this idea, Grbeša *et al*. reported that AHCY and AHCYL1 co-immunoprecipitated in HEK293T cells, but endogenous interactions were not robust, and functional effects were not examined (49). Parkhitko *et al*. similarly suggested that *Drosophila* AHCYL1 directly binds and inhibits AHCY to extend lifespan but demonstrated only a weak unidirectional co-immunoprecipitation of overexpressed AHCY and AHCYL1 in cultured S2 cells (50). We were unable to observe an interaction between AHCY and AHCYL1 using yeast two-hybrid or co-immunoprecipitation (29). Moreover, the *AHCYL1* RNAi lines used in the Parkhitko study significantly decreased *AHCY* transcript levels, despite not having overlapping RNAi target sequences (29). These findings may indicate a potential transcriptional cross-talk model between *AHCY* and *AHCYL1*, in which AHCYL1 regulates AHCY expression, but this has not been examined. Overall, the current body of evidence suggests that while an AHCY:AHCYL1 interaction is structurally plausible, this may represent a weak or transient association with limited regulatory significance.

## Properties of AHCY as a conserved enzyme in fundamental cellular metabolism

### The metabolic role of AHCY

**Figure 7:**
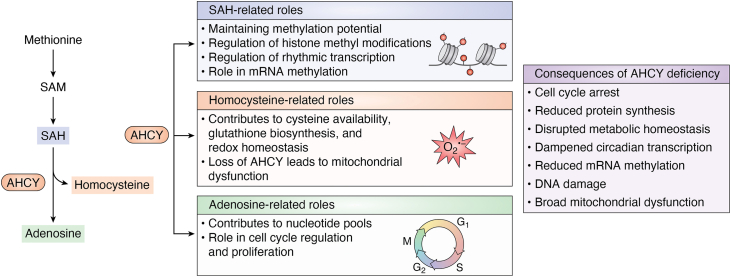
**Roles of AHCY in metabolism and consequences of AHCY deficiency**. AHCY converts SAH to adenosine and homocysteine, and these three metabolites shape distinct biological processes. SAH levels influence cellular methylation capacity and chromatin-associated methyl marks. Adenosine supports nucleotide balance and cell cycle progression. Homocysteine contributes to cysteine production, glutathione biosynthesis, and redox balance. Together these processes connect AHCY to transcriptional regulation, cell growth, and mitochondrial health. Loss of AHCY disrupts these processes and results in widespread defects that include impaired protein synthesis, altered metabolic and circadian regulation, DNA damage, and mitochondrial dysfunction.

In higher eukaryotes, AHCY is the sole enzyme to perform the reversible catalysis of SAH into adenosine and homocysteine (33). Because each of these reaction components exerts distinct downstream effects, the functional roles of AHCY can be broadly organized into three categories: SAH-related, adenosine-related, and homocysteine-related processes (Fig. 7). These categories provide a framework to explore how AHCY influences diverse cellular pathways, including stem cell differentiation, cellular proliferation, chromatin modifications and accessibility, transcription, and nucleic acid processing (1).

In addition to genetic manipulation models, much of our understanding of AHCY function has been derived from small-molecule inhibitors (Table 2). Most of these are type I inhibitors that bind at or near the active site in the closed conformation, often competing with SAH and interacting with the NAD^+^ molecule (51). By mimicking the transition state, the inhibitors stabilize the catalytically active form of the AHCY. Although no *bona fide* type II inhibitors have been identified, mutations such as K426R create a type II–like effect by locking AHCY in an inactive NADH-bound state (43). The following sections integrate how these inhibitors together with genetic models have revealed the biological roles of AHCY.

**Table 2:** AHCY type I inhibitors

Inhibitor	Structure	Binding/Mechanism	Most common applications	Key references
3-Deazaneplanocin A (DZnep)		Adenosine analog; SAH-competitive binds active site of AHCY to prevent catalysis	Epigenetics, cancer biology, and circadian studies	(65, 67, 68)
Adenosine dialdehyde (AdOX)		Irreversible inhibitor; reacts with lysine residues to stabilize enzyme-bound SAH	Global methylation inhibition and differentiation studies	(86, 87, 88)
Sinefungin		SAM analog; competes at methyl donor site, not AHCY-specific	Methyl cycle disruption	(89)
3-Deazaadenosine (3-DA)		SAM analog; competes at methyl donor site	Transmethylation studies and viral replication	(87, 90, 91)
3-Deazaaristeromycin & Analogs		Carbocyclic adenosine analogs; mimic SAH and adenosine binding to inhibit AHCY	Mechanistic and structural studies	(92, 93, 94, 95)

Table describing widely used AHCY inhibitors (structures from PubChem) with details on binding mechanisms.

### SAH-related roles: Chromatin and methylation capacity

SAH is a potent feedback inhibitor of nearly all cellular methyltransferases (52, 53, 54, 55), making its removal by AHCY essential for sustaining methylation potential and thereby regulating chromatin state and transcriptional programs. Due to the importance of maintaining low SAH levels, AHCY has emerged as a critical regulator of transcription and chromatin accessibility dynamics. In pluripotent stem cells, Aranda *et al*. demonstrated that AHCY associates with chromatin, specifically at the 5′ UTR and transcriptional start site of genes involved in ribosomal biogenesis and mRNA processing using DNA-mediated chromatin pull-down (56). A majority of these binding sites are on highly expressed genes marked by histone modifications associated with transcriptional activation such as H3K4me3, H3K36me3, and H3K27ac with minimal levels of repressive marks like H3K27me3 (56). Genetic downregulation of AHCY led to cell cycle arrest at G1/S phases, reduced protein synthesis, and global downregulation of ribosomal proteins (56, 57).

AHCY is a direct transcriptional target of OCT4 that is essential for maintaining pluripotency in mouse embryonic stem cells (58). Jiang *et al*. showed that downregulation of AHCY led to failure in silencing OCT4 expression, suggesting that AHCY is necessary for the exit of pluripotent states (58). AHCY deficiency disrupted metabolic homeostasis, led to decreased ATP, and increased reactive oxygen species *via* decreased production of cystathionine, glutathione, and serine (58). Additionally, epigenetic marks including H3K4me3 and H3K27me3 were decreased at transcriptional start sites as well as expression levels of the methyltransferase EZH2 upon AHCY downregulation (58). Furthermore, AHCY was identified to interact with both EZH2 and SUZ12, core proteins of the polycomb repressive complex 2, suggesting that AHCY likely maintains H3K27me3-mediated gene repression and H3K4me3-dependent gene activation by these interactions (58).

Outside of histone methylation, AHCY also influences DNA methylation. This occurs through an interaction with DNA methyltransferase 1 (DNMT1) during S-phase, where AHCY colocalizes with DNMT1 during replication (59). Ponnaluri *et al*. demonstrated that AHCY forms a complex with DNMT1 *in vivo* and that this association is enriched during DNA synthesis. Futhermore, *in vitro* assays showed that the addition of recombinant AHCY enhanced DNMT1 catalytic activity. This effect was observed not only with WT AHCY, but also with AHCY mutant variants. Inhibition or depletion of AHCY in cells reduced overall DNA methylation, whereas overexpression promoted hypermethylation, supporting a direct role for AHCY in modulating DNMT1 function (59). Together, these findings indicate that AHCY supports DNMT1-dependent methylation not solely *via* SAH levels, but potentially also through protein–protein interactions.

AHCY may also have roles outside of development such as regulating rhythmic transcription and circadian chromatin remodeling. Greco *et al*. identified a time-dependent protein–protein interaction between AHCY and the core circadian transcription factor BMAL1 in mouse liver (60). AHCY binds BMAL1 at its PAS-B domain, which is required for the dimerization of CLOCK:BMAL1 and transcriptional activity (60). Genetic manipulation and pharmacological inhibition of AHCY dampened circadian transcription in mouse embryonic fibroblasts and reduced BMAL1 occupancy at target promoters including two known output genes *Dpd* and *Per2* (60). Moreover, rhythmic deposition of H3K4me3 at clock gene promoters was nearly abolished upon AHCY inhibition, indicating that AHCY contributes to temporal dynamics of chromatin modifications necessary for circadian gene expression (60). Importantly, downregulation of AHCY did not change total protein abundance of BMAL1, suggesting that AHCY is required for its recruitment rather than protein stability (60).

Whereas these first three studies implicated AHCY in active histone methylation, Campbell *et al*. showed that AHCY is required for repressive histone modifications in Müller glia-derived progenitor cells following injury in the retina (61). Inhibition of AHCY was accompanied by a significant reduction in the repressive histone mark H3K27me3, along with the downregulation of genes involved in chromatin remodeling including *EZH2* across multiple retinal cell types using single-cell transcriptomic and epigenomic profiling approaches (61). In contrast to these findings, our study in *Drosophila* photoreceptors revealed increased H3K27me3 upon AHCY knockdown, yet these changes did not correspond with the suppression of light stress-induced gene expression, suggesting that AHCY-dependent transcriptional regulation does not directly reflect changes in H3K27me3 or H3K4me3 levels (29). Thus, it remains unclear whether histones are necessarily the relevant targets of AHCY activity that mediates changes in gene expression.

Emerging evidence suggests that mRNA may instead represent a critical substrate for mediating transcriptional responses (62, 63). Several studies have implicated RNA methylation as a major target of AHCY activity, particularly through its role in nuclear RNA metabolism and mRNA processing. To elucidate the involvement of AHCY in nuclear RNA metabolism, Radomski *et al*. conducted a series of studies using *Xenopus laevis* in which AHCY exhibited dynamic subcellular localization during embryogenesis, suggesting functions beyond its canonical enzymatic activity (62). AHCY was found to translocate to nuclear fractions during G1/2 phases of the cell cycle, coinciding with heightened transcriptional activity (62). Site-directed mutagenesis studies highlighted that the N-terminal region of AHCY is necessary for nuclear localization (62). Mutations to the C terminus led to a loss of a conserved helical domain resulting in greater than 50% of the AHCY protein being unable to translocate, remaining instead in the cytoplasm (62). Furthermore, immunohistological analyses revealed that AHCY and RNA Pol II colocalized on active transcriptional loops, suggesting a potential role in RNA synthesis (62). In a cultured cell model, AHCY pharmacological inhibition led to reduced cap methylation and synthesis of polyadenylated RNA, implicating AHCY in both cap methylation of pre-mRNA and transcriptional elongation processes (62).

Expanding on these findings, Radomski *et al*. explored the interplay between AHCY and capping methyltransferases *in vivo* and *in vitro* (63). Using protein–protein interaction assays, AHCY was found to associate with the capping methyltransferase enzyme, mRNA(guanine-N7)-methyltransferase (CMT), *via* the C terminus of AHCY without any additional cofactors (63). CMT is responsible for the final step in mRNA capping, suggesting a collaborative role in mRNA cap formation between AHCY and CMT. Moreover, inhibition of AHCY led to the premature export of heterogeneous nuclear RNA, indicating that AHCY plays an important role in proper RNA processing (63). Given that CMT interacts with the phosphorylated C-terminal domain of RNA Pol II, it is postulated that the interaction between AHCY and CMT may facilitate its nuclear import and positioning within transcriptionally active domains (63).

Supporting these observations from *Xenopus*, Fernandez-Sanchez *et al*. demonstrated that AHCY is essential for MYC-induced mRNA cap methylation in mammalian cells (64). MYC is a well-characterized transcription factor that when overexpressed led to increased AHCY levels and promoted mRNA cap methylation and protein production (64). Supporting this finding, pharmacological inhibition and genetic manipulation results in decreased mRNA cap methylation, protein production, and cell proliferation (64). These findings reinforce the idea that AHCY plays a broader role outside of 1C metabolism.

Pharmacological inhibitors have been particularly informative in this context. 3-Deazaneplanocin A (DZnep), a carbocyclic adenosine analog (lacking the oxygen atom in the sugar group) that mimics SAH, has been shown to reduce global methylation capacity and alter histone modification patterns (65, 66). DZnep was originally developed as a potent inhibitor of AHCY (67, 68). DZnep treatment leads to intracellular accumulation of SAH and thus broadly decreases SAM-dependent methyltransferase activity (65). Intriguingly, DZnep has also been shown to decrease protein levels of polycomb repressive complex 2 components such as EZH2 leading to decreased levels of H3K27me3; however, Western blot analysis indicates that DZnep broadly inhibits histone methylation and is not specific for H3K27me3 (65). Sinefungin, though not AHCY-specific, also disrupts methylation-dependent pathways and has been used to probe the methyl cycle (69). These studies highlight how small molecule inhibition of AHCY directly impacts chromatin methylation and transcriptional programs.

Collectively, these studies highlight the multifaceted roles of AHCY in gene regulation, extending beyond its canonical activity in SAH catalysis to encompass both chromatin and RNA metabolism. AHCY influences permissive and repressive chromatin states in a context-dependent manner while also contributing to mRNA cap methylation, nuclear RNA processing, and interactions with the transcriptional machinery, positioning it as a versatile regulator of transcriptional programs essential for processes such as circadian transcription and retinal regeneration.

### Adenosine-related roles: Cell cycle, proliferation, and pluripotency

Adenosine, the second product of AHCY catalysis, contributes to nucleotide pool balance and purinergic signaling, enabling AHCY activity to influence DNA synthesis, cell cycle progression, and cellular proliferation. In hepatoma cells, pharmacological inhibition of AHCY by the adenosine analog DZnep induces cellular senescence, which is characterized by G2/M cell cycle arrest coupled with the upregulation of checkpoint inhibitors including cyclin-dependent kinases p16, p21, and p27 at both protein and transcript levels (70). Wu *et al*. identified this senescence as being mediated *via* the activation of the DNA damage response pathway because upstream and downstream factors including pATM, H2AX, and pCHK1/2 were upregulated upon inhibitor treatment (70).

Similar results were observed in a genetic manipulation model where *AHCY* knockdown in HepG2 cells led to adenosine depletion causing an imbalance of deoxyribonucleotide triphosphate pools, specifically decreasing dATP and cell cycle arrest at G1/S phases (57). This imbalance resulted in the stalling of replication forks, which is associated with replication stress and activation of DNA damage response pathways (57). Consequently, there was an accumulation of double-stranded breaks supported by increased γH2AX levels and increased protein abundance of cell cycle proteins including serine/threonine-protein kinase CHK1 and CHK2, which are implicated at the G2 checkpoint (57). Importantly, supplementation with adenosine in AHCY-deficient HepG2 cells showed partial restoration of deoxyribonucleotide triphosphate pools, alleviated replication stress, reduced DNA damage markers, and resumed normal cell cycle progression (57). The ability to rescue these phenotypes emphasizes the critical role of AHCY in maintaining nucleotide and cellular homeostasis.

Early studies with 3-deazaadenosine, an adenosine analog inhibitor modified at position 3 of the adenine ring where the nitrogen is replaced with a carbon, decreased AHCY activity by over 90% *in vitro* and elevated SAH levels in hepatocytes (71). Together, with the DZnep results above, these findings emphasize how pharmacological inhibitors of AHCY disrupt nucleotide balance, trigger DNA damage, and interfere with proliferation, providing complementary evidence to genetic manipulation models. Collectively, these findings highlight AHCY’s role in regulating cell cycle progression, maintaining pluripotency, and ensuring genomic stability across various cell types.

### Homocysteine-related roles: Redox balance and metabolism

Homocysteine, one of the two products generated by AHCY catalysis, serves as a critical branch point between the methionine and transsulfuration pathways, directly linking AHCY activity to cysteine availability, glutathione biosynthesis, and cellular redox homeostasis. Rowland *et al*. demonstrated that genetic downregulation of *AHCY* in glioblastoma cells led to increased lipid peroxidation and impaired mitochondrial respiration indicative of oxidative stress (72). Additionally, *AHCY* deficiency resulted in reduced basal respiration, ATP production, and protein leakage using Seahorse analysis (72). These findings suggest that downregulation of *AHCY* causes broad mitochondrial dysfunction. These findings were recapitulated using pharmacological inhibitors including AG-270 and aristeromycin (72). Notably, supplementation with either GSH or SAM was sufficient to restore mitochondrial function in AHCY-inhibited cells (72). Because homocysteine is a precursor molecule required for GSH production, the ability of GSH to rescue function likely reflects this metabolic connection. Global metabolomic analysis revealed severe disruption in antioxidant production pathways, fatty acid metabolism, and nucleotide biosynthesis (72).

In *Drosophila*, our work showed that oxidative inhibition of AHCY at C195 resulted in increased SAH levels and a marked decrease in the GSH:GSSG ratio, indicative of diminished antioxidant capacity. Although homocysteine was not directly measured, the depletion in reduced glutathione is consistent with reduced flux through the transsulfuration pathway, in which homocysteine serves as the precursor to cysteine for glutathione biosynthesis (29, 73). Despite this metabolic shift, transient inhibition of AHCY in the eye was neuroprotective, as reflected in reduced photoreceptor degeneration upon blue light exposure, which we attributed to the suppression of light stress–induced gene expression programs (29).

Together, findings from Stanhope *et al*. and Rowland *et al*. highlight the central role of AHCY in redox homeostasis, mitochondrial function, and cellular stress responses. Although inhibition of AHCY disrupts antioxidant capacity and respiration, our *Drosophila* studies show that transient inhibition in the eye can provide short-term neuroprotection, underscoring a context-dependent role for AHCY in linking metabolism to cell survival. These studies emphasize the multifaceted role of AHCY at the intersection of metabolism and gene regulation, where its activity can either compromise or preserve cellular function depending on context and duration.

### Translational insights from inhibitors

Beyond serving as experimental probes, AHCY inhibitors have highlighted the enzyme’s translational relevance. Neplanocin A, a cyclopentenyl adenosine analog, impaired viral RNA methylation and blocked replication of vaccinia virus with minimal host toxicity (74). DZnep and Neplanocin A also demonstrated anti-HIV activity by reducing viral p24 antigen levels (75). Another well-characterized analog, 3-deazaaristeromycin, has been widely used in mechanistic studies and displayed broad antiviral activity through disruption of viral RNA methylation (74, 76). In cardiovascular studies, inhibition of AHCY with adenosine dialdehyde in mice induced endothelial dysfunction, impaired vascular relaxation, and increased oxidative stress (77). These findings link elevated SAH levels and impaired AHCY activity with cardiovascular pathology. Together, these studies extend the biological roles of AHCY beyond core metabolism and gene regulation into cancer, viral infection, and cardiovascular disease, illustrating how inhibition-based approaches have not only uncovered mechanistic insights but also opened translational opportunities.

### AHCY deficiency and its pathophysiological consequences

**Figure 8:**
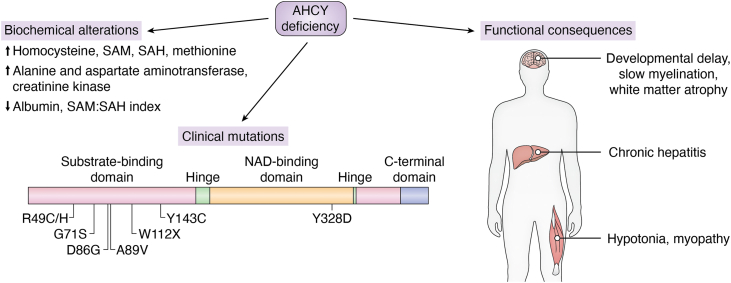
**Schematic summarizing the biochemical and functional consequences of AHCY deficiency metabolic disorder**. Biochemical alterations include elevated homocysteine, *S-*adenosylmethionine (SAM), *S-*adenosylhomocysteine (SAH), and methionine, as well as increased alanine and aspartate aminotransferase and creatine kinase levels, with decreased albumin and the SAM:SAH index. Reported pathogenic mutations are distributed across the substrate-binding domain (W112X, Y143C, R49C/H, D86G, G71S, and A89V) and NAD + -binding domain (Y328D). Functionally, AHCY deficiency symptoms include developmental and psychomotor delays, slow myelination, and white matter atrophy in the brain, chronic hepatitis in the liver, and hypotonia and myopathy in muscle tissues.

Pathogenic mutations in *AHCY* cause a rare autosomal recessive metabolic disorder known as AHCY deficiency. To date, there are fewer than two dozen cases reported worldwide, highlighting that AHCY is indispensable for human viability, and even partial reductions in activity lead to marked metabolic and neurological consequences (78, 79, 80). The earliest described patients presented with neonatal hypotonia, psychomotor delay, myopathy, and hepatic dysfunction accompanied by a biochemical signature of hypermethioninemia, elevated SAH and SAM, and increased serum creatine kinase (Fig. 8) (80, 81).

Functional studies revealed that the allelic missense variants R49C and D86G almost completely abolish catalytic activity (78). The R49C mutant forms intermolecular disulfide bonds that render the enzyme inactive, while D86G substitution promotes aggregation and loss of function due to removal of a negative charge. In the D86G mutant, enzymatic activity can be partially restored by the reintroduction of a negatively charged residue such as D86E (78). Supporting this, subsequent reports identified additional pathogenic variants including R49H, G71S, Y143C, Y328D, and a premature stop codon W112X, which cluster near the substrate- and NAD^+^-interacting regions (79, 81). Phenotypic severity varies, with some individuals displaying early lethal hepatic failure while others showed milder, adult-onset, or even asymptomatic forms (79, 81, 82). This variability likely reflects differences in residual enzymatic activity. For example, the R49H mutant retains partial function and has been linked to delayed-onset or subclinical presentation (81, 82). Recent work further demonstrated global DNA hypermethylation and locus-specific changes in affected patients, consistent with SAH accumulation inhibiting methyltransferases and disrupting cellular methylation homeostasis (83).

Complementary evidence from model organisms emphasizes the essential developmental requirement for AHCY. Complete *AHCY* knockout in mice results in early embryonic lethality, with homozygous null embryos failing to progress due to global disruption of methylation-dependent processes (84, 85). These findings support AHCY’s central role in maintaining methylation potential during cellular proliferation and differentiation in early development (56, 57, 86). Together, the clinical human genetic data and knockout models support that both total and partial loss of AHCY activity perturbs 1C metabolism and methylation-dependent regulation. These insights reinforce the need for careful therapeutic titration when considering pharmacological inhibition.

## Regulatory mechanisms governing AHCY activity

### AHCY regulation by posttranslational modifications

Posttranslational modifications have emerged as key regulatory mechanisms for modulating AHCY enzymatic activity. In *M*. *tuberculosis*, AHCY is a direct substrate of the serine/threonine kinase PknB (24). PknB phosphorylates AHCY *in vitro* and *in vivo* at T219-221, located within the substrate-binding domain adjacent to the NAD^+^-binding pocket (24). Threonine phosphorylation decreases AHCY’s affinity for NAD^+^ without altering *V*_*max*_, suggesting this phosphorylation modulates catalytic function primarily *via* cofactor binding rather than catalytic efficiency or turnover rate (24). Importantly, a phospho-deficient triple AHCY mutant (T219A/T220A/T221A) was catalytically inactive in the hydrolytic direction, emphasizing the essential role of these residues for SAH hydrolysis and NAD^+^ binding (24). Database mining using PhosphoSite Plus revealed a conserved tyrosine phosphorylation event at Y193 in both human and mouse AHCY. Despite its conservation and frequent detection in phosphoproteomic datasets, no studies to date have investigated how phosphorylation at this residue affects AHCY structure or enzymatic activity (96, 97).

In addition to phosphorylation, AHCY is regulated by lysine acetylation at two conserved C-terminal residues, K401 and K408 (25). The functional and structural consequences of these residues were explored by Wang *et al*. using site-specific acetylation mutants to generate either mono- or di-acetylated AHCY (25). All AHCY acetylated variants displayed approximately threefold decreases in catalytic efficiency compared to WT AHCY (25). High-resolution structure analysis revealed that acetylation disrupted nearby sidechains and key hydrogen-bonding networks. The effected residues spanned the substrate-binding domain (N27), the NAD^+^-binding domain (D293), and the C-terminal domain (D422) (25). These alterations were predicted to destabilize the local interactions necessary for enzyme activity and transition between conformations. To support this idea, point mutants asparagine to lysine (N27K) and aspartic acid to alanine (D293A) were generated and found to have similar decreases in catalytic efficiency and increases in *K*_*m*_ values, recapitulating the observed effect of mono- and di-acetylation (25). Together, these findings suggest that acetylation governs AHCY activity by altering intramolecular hydrogen bonds within the active site and cofactor-binding regions.

Besides phosphorylation and acetylation, other posttranslational modifications such as β-hydroxybutyrylation, 2-hydroxyisobutyrylation, and O-GlcNAcylation have expanded the regulatory network of AHCY activity (30, 98, 99). β-hydroxybutyrylation is a modification associated with the metabolic adaptation response to nutrient stress. AHCY undergoes β-hydroxybutyrylation in mouse embryonic fibroblasts at 4 lysine residues K188, K204, K389, and K405, which are located near the interface between the C-terminal loops in adjacent subunits that coordinate the substrate- and cofactor-binding domains (98). Based on the location of these modifications, β-hydroxybutyrylation may interfere with cofactor binding or proper substrate positioning. K188 is known to participate in hydrogen-bond interactions with the C-terminal tails between the homodimers, and it is likely that a β-hydroxybutyrylation modification would introduce a positive charge potentially disrupting these hydrogen-bonding events (98). Indeed, β-hydroxybutyrylation at these residues led to decreased AHCY activity and increased SAH levels (98). To further support this finding, AHCY lysine to arginine mutants were generated and resulted in a significant decrease in enzymatic activity (98). Importantly, the K188R AHCY mutant completely inactivated the enzyme, reinforcing the importance of K188 in both structural and functional contexts.

2-hydroxyisobutyrate is a precursor molecule for the synthesis of 2-hydroxyisobutyrylation, which is a modification associated with active gene expression (99). Huang *et al*. performed one of the first global profiling studies to determine 2-hydroxyisobutyrylation substrates in human cells (99). Interestingly, a majority of the identified proteins were highly enriched in metabolic pathways. 2-hydroxyisobutyrylation was detected on AHCY at K186 located in the cofactor-binding domain (99). Although there have been no studies to evaluate the functional consequence of 2-hydroxyisobutyrylation at K186, given its proximity to the catalytic core, this modification may decrease activity and disrupt residue interactions similar to the observed effect of β-hydroxybutyrylation.

O-GlcNAcylation is a monosaccharide modification on serine and threonine residues that occurs in response to nutrient availability linking metabolic sensing by nucleocytoplasmic proteins to transcriptional and differentiation processes (30). Zhu *et al*. identified AHCY possessing O-GlcNAcylation modification on T136 using chemoenzymatic labeling (30). To determine if O-GlcNAcylation at T136 is important for enzymatic function, the residue was mutated to an alanine (T136A) resulting in decreased AHCY activity in mouse embryonic stem cells (30). This decrease in enzymatic activity was also coupled with decreased cell proliferation and impaired maintenance of pluripotency (30). Additionally, the pharmacological elevation of O-GlcNAcylation levels led to increased tetramer formation of WT AHCY, but not the T136A mutant, indicating that O-GlcNAcylation at this site is required for stabilizing oligomerization (30). These findings suggest that this modification may promote AHCY activity by enhancing its structural conformation and cofactor binding.

### Regulation *via* oxidative modifications

Although oxidative modifications are broadly classified as posttranslational modifications, they are often considered mechanistically distinct. Notably, redox regulation of AHCY has been shown to impact its enzymatic activity in both *in vitro* and *in vivo* settings in *Drosophila*. Our recent study demonstrated that AHCY undergoes an oxidative modification at a conserved C195 resulting in inhibition of its catalytic function (29). C195 is positioned near the NAD^+^ nicotinamide ring and is within hydrogen-bonding distance of H353. Thus, oxidative modification at C195 may result in altered positioning of nearby residues including H353, D190, and K185 leading to changes in catalysis. Interestingly, C195 is conserved in all eukaryotic AHCY homologs examined except *C*. *elegans* and is absent in bacterial homologs, suggesting that redox regulation at this site may be a feature that evolved in most animals to enable oxidative control over methylation capacity and metabolic flux.

Collectively, these findings reveal that AHCY is regulated by diverse posttranslational modifications that integrate metabolic sensing with gene regulation through changes in activity, structure, and protein interactions. Many of these modifications are species-dependent. Phosphorylation by PknB has so far only been described in *M*. *tuberculosis*, whereas acetylation, β-hydroxybutyrylation, 2-hydroxyisobutyrylation, and O-GlcNAcylation have been characterized in mammalian systems, where they alter catalytic efficiency and tetrameric stability. Oxidative regulation at C195 is conserved across higher eukaryotes but absent in *C*. *elegans* and bacteria, suggesting that redox sensitivity arose later in evolution to enable oxidative control over methylation capacity. Together, these observations highlight that while posttranslational modifications universally regulate AHCY, the specific mechanisms vary between species to meet distinct cellular and environmental demands.

### Regulation *via* direct metabolite binding

AHCY activity is also regulated by small molecules involved in 1C metabolism. 5′-MTA, a byproduct of polyamine biosynthesis within the 1C metabolic network, can directly suppress AHCY activity. Early biochemical studies demonstrated that micromolar concentrations of MTA rapidly inactivate AHCY *in vitro*, reducing enzymatic activity by as much as ∼70 to 80% within minutes of incubation. This inactivation is irreversible, as activity could not be recovered by extensive dialysis, consistent with a suicide substrate–like process in which the catalytic cycle converts MTA into a reactive intermediate that covalently disables the enzyme (100, 101). Ragione *et al*. further showed that closely related thioadenosine derivatives, including 5′-isobutylthioadenosine, produced comparable levels of rapid and irreversible inactivation, reinforcing that the 5′-thio substituent engages the catalytic machinery to trap the enzyme (101). Yuan *et al*. demonstrated that fluorinated 5′-methylthioadenosine derivatives resulted in time-dependent inactivation accompanied by the reduction of the enzyme-bound NAD^+^ and release of fluoride (102). As with native MTA, the activity loss was irreversible and not reversed by dialysis (102). These studies establish that micromolar amounts of MTA and related thioadenosine analogs can drive substantial, rapid, and irreversible inactivation of AHCY. Although precise inhibition values differ depending on the assay system and substrate analog used, multiple reports document ∼80% loss of activity over short timeframes, underscoring the potency of MTA as a regulatory metabolite (100, 101, 102, 103).

Taken together, the studies summarized in this review highlight that AHCY is more than a simple housekeeping enzyme involved in 1C metabolism. AHCY activity connects SAH turnover to adenosine- and homocysteine-mediated pathways, thereby linking nucleotide balance, redox homeostasis, and methylation capacity (Figs. 2 and 7). Structural analyses highlight a highly conserved core enzyme with species-specific variations in oligomeric state, NAD^+^ pocket accessibility, and C-terminal dynamics that shape catalytic efficiency and cofactor control. Kinetic comparisons reveal that while the catalytic mechanism is preserved, organisms have adapted AHCY’s substrate affinity, inhibition sensitivity, and turnover rates to meet distinct metabolic and regulatory demands. Regulation by posttranslational modifications further integrates nutrient sensing, redox signaling, and transcriptional control, with modification patterns differing across bacteria, protozoa, and higher eukaryotes. Collectively, these layers of metabolic function, structural specialization, and regulatory flexibility emphasize that AHCY operates as a central regulatory node at the intersection of metabolism, gene regulation, and stress adaptation. Given that perturbations in AHCY activity are linked to cancer, neurodegeneration, cardiovascular disease, and pathogen replication, understanding its regulation across species not only illuminates fundamental biology but also identifies opportunities for therapeutic intervention.

## Conflicts of interest

The authors declare that they have no conflicts of interest with the contents of this article.
